# Pannexins in the musculoskeletal system: new targets for development and disease progression

**DOI:** 10.1038/s41413-024-00334-8

**Published:** 2024-05-06

**Authors:** Yan Luo, Shengyuan Zheng, Wenfeng Xiao, Hang Zhang, Yusheng Li

**Affiliations:** 1 Department of Orthopedics, Xiangya Hospital, Central South University, Changsha, Hunan 410008 China; 2 National Clinical Research Center for Geriatric Disorders, Xiangya Hospital, Central South University, Changsha, Hunan 410008 China; 3 Department of Clinical Medicine, Xiangya Medicine School, Central South University, Changsha, Hunan 410008 China; 4 Shenzhen Institute of Advanced Technology, Chinese Academy of Sciences, Shenzhen, 518055 China

**Keywords:** Bone, Pathogenesis, Osteoporosis, Osteogenesis imperfecta

## Abstract

During cell differentiation, growth, and development, cells can respond to extracellular stimuli through communication channels. Pannexin (Panx) family and connexin (Cx) family are two important types of channel-forming proteins. Panx family contains three members (Panx1-3) and is expressed widely in bone, cartilage and muscle. Although there is no sequence homology between Panx family and Cx family, they exhibit similar configurations and functions. Similar to Cxs, the key roles of Panxs in the maintenance of physiological functions of the musculoskeletal system and disease progression were gradually revealed later. Here, we seek to elucidate the structure of Panxs and their roles in regulating processes such as osteogenesis, chondrogenesis, and muscle growth. We also focus on the comparison between Cx and Panx. As a new key target, Panxs expression imbalance and dysfunction in muscle and the therapeutic potentials of Panxs in joint diseases are also discussed.

## Introduction

A plethora of mechanical and interactive signals exist in the musculoskeletal system, necessitating tight cellular communication to maintain the integrity of cellular structure and function.^1^ Such communication channels as gap junctions (GJs), hemichannels (HCs), and ion channels respond directly or indirectly to the coordinated cells of these extracellular signals. Two crucial channel component proteins, the connexin (Cx) (particularly Cx43) and pannexin (Panx), are abundantly expressed in the extensively interconnected bone network that is formed by osteoblasts and osteocytes.^2,3^ This osteogenic network plays a pivotal role in the process of bone responding to various stimuli such as mechanical loading, hormone, and growth factor signals, thereby regulating bone quality.^4^ In addition to their presence in the bone network, Panxs and Cx43 have also been reported to be expressed in osteoclasts, undifferentiated muscle precursor cells, mature muscle cells, and chondrocytes.^5,6^ Currently, Panxs are beginning to come into focus like Cx43, with increasing evidence suggesting that Panxs contribute significantly to the function of these cells.

Panchin et al. were the first to identify the Panx family in mammalian genomes, which includes Panx1, Panx2, and Panx3.^7^ Panxs are capable of forming nonselective large-pore membrane channels, serving as a bridge between the intracellular and extracellular environments.^8,9^ These channels facilitate the exchange of ions and small molecules between adjacent cells and between cells and the extracellular matrix. Examples of these ions or molecules include K^+^, Cl^-^, Ca^2+^, glutamate, adenosine triphosphate (ATP), and inositol triphosphate 3 (IP3).^10–14^ Although Panxs do not share significant homology with Cxs,^15,16^ they are considered to possess many functional characteristics similar to Cxs.^17^

Currently, the role of Panxs in the musculoskeletal system has not been thoroughly studied. This review will provide an overview of the basic structure and functions of Panxs in the musculoskeletal system. We will also discuss the key roles of Panxs in osteoblasts, osteoclasts, osteocytes, chondrocytes, tendon, and ligament. Particular attention will be paid to the newly discovered roles of Panxs in osteogenesis, chondrogenesis, and myoblast differentiation. Additionally, we will compare Panxs with Cxs (with Cx43 as a representative), and discuss the latest research on Panxs and their potential as new therapeutic targets.

## Expression, basic molecular structure and function of Panxs

### Expression of Panxs

The Cx family, with nearly 21 members, has been well defined and characterized. They are expressed in the musculoskeletal system, including in bone, cartilage, skeletal muscle, and synovium, with Cx43 being the most widely expressed connexin in these tissues.^18^ In contrast, the Panx family has been not as well characterized, and only three members (Panx1–3) have been found to be ubiquitously expressed. Panx1 has been reported to be widely expressed at both the mRNA and protein levels in many tissues, including the eye, liver, kidney, and bone.^19–21^ Panx1 has also been detected in the skeletal muscle system of mice, rats, and humans,^22–24^ including in differentiated myoblasts,^25^ myotubes,^26^ and myofibers.^24,27^ Furthermore, Panx1 expression has been detected in the periodontal ligament,^28^ but not in the tendon. On the other hand, Panx2 mRNA appears to be highly enriched in the central nervous system, and the Panx2 protein (664 amino acids, 73.3 kD) is mainly located in the cytoplasmic compartment.^29^ Panx3 mRNA and protein have primarily been reported in the skin, chondrocytes, osteoblasts, and synovial fibroblasts.^20,22,30,31^ The Panx3 protein is also expressed in skeletal muscle of mouse, rat, and human.^25^ However, recent studies have found that Panx2 expression is not restricted to the central nervous system, but has a more ubiquitous expression than previously predicted^32^ and has been detected in osteoblasts.^21^ In addition to Panx1 and Panx3, the Panx2 protein has been found to express in mouse skeletal muscle^32^ and in human primary myotubes.^33^

### Basic molecular structure of Panxs

Several recent studies have utilized cryo-electron microscopy (cryo-EM) to discover that Panx1 (426 amino acids, 47.6 kD) channels form heptameric assemblies, each of which demonstrates the typical topology of four transmembrane proteins which is also seen in other large pore channels, such as Innexin and Cx.^34–39^ Panx2, which was once proposed to form an octamer,^40^ is now revealed by the latest cryo-EM structure to have seven identical prototypes forming a heptamer. Each protomer is assembled symmetrically around the central axis of the channel pore.^41^ The structure of Panx3 (392 amino acids, 43 kD), however, has not yet been defined, and its oligomeric state remains unstudied.

**Fig. 1:**
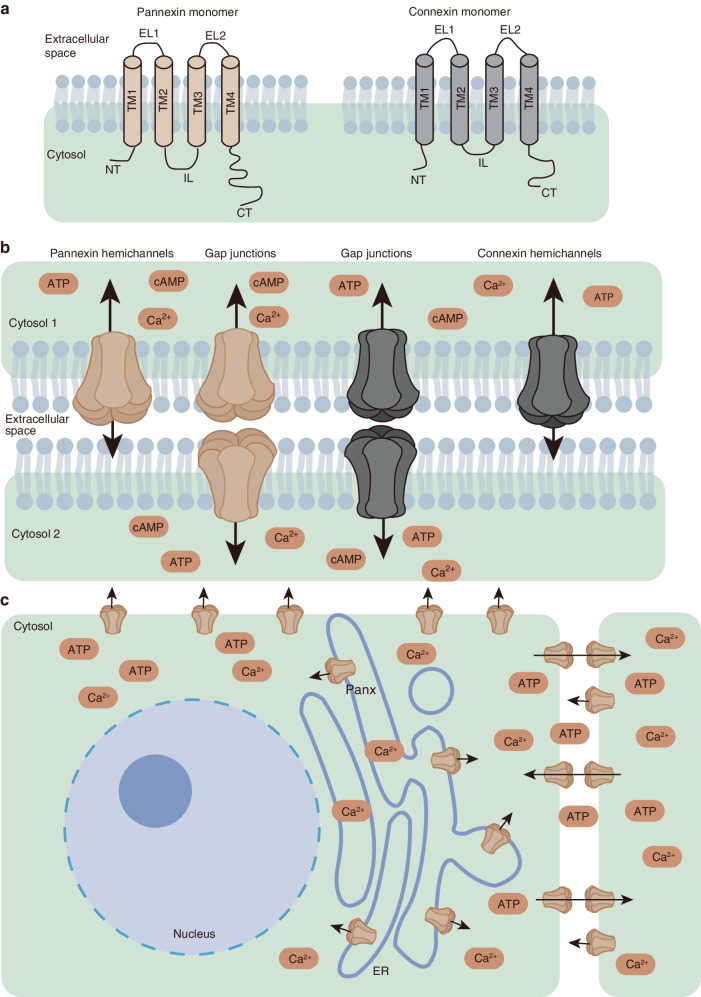
Structure of GJ proteins. **a** Prototypic secondary structure of GJ proteins (Panxs and Cxs). Each Panx and Cx monomer consists of 2 extracellular loops, 4 transmembrane domains, and 1 intracellular loop. **b** GJ and hemichannels assembled by Panxs and Cxs subunits. Panx subunit function as channels, termed HCs (heptamer), which allow the exchange of small molecules and ions. Cx HCs (hexamer) are similar. What is currently certain is that two Cx HCs can combine to form GJs, but whether Panx HCs can form GJs is still questionable. **c** Schematic diagram of Panxs positioning and channel functions. Panxs can aggregate to form single-membrane channels, which are located on the cell surface and ER and are responsible for the intracellular and extracellular exchange of metabolites and ions such as ATP and Ca^2+^ and the release of Ca^2+^ in the ER (not include Panx1). (ER endoplasmic reticulum, ATP adenosine triphosphate, cAMP cyclic adenosine monophosphate, GJ gap junction, HCs hemichannels.)

The Panx1 protomer structure contains four transmembrane helices (TM1–TM4), two extracellular loops, and one intracellular loop. The amino terminus (NT) and carboxyl terminus (CT) of the protein are located on the cytoplasmic side facing the center of the channel, where the NT region is short and completely invisible in the structure, whereas the CT domain contains two α-helices (ICH3 and ICH4).^35,42–44^ The intracellular loop contains two α-helices (ICH1 and ICH2) and a disordered region consisting of 2 residues.^35^ In comparison, each protomer of Panx2 can be divided into three domains: extracellular domain (ECD), transmembrane domain (TMD) and intracellular domain (ICD). The ECD consists of 2 extracellular loops, and the TMD consists of four helices (TM1–TM4). The ICD is a helix-rich structure consisting of a cytoplasmic loop connecting TM2 and TM3 and the CT residues following TM4.^41^ Within the Panx family, Panx1 and Panx3 exhibit high structural similarity, while Panx2 has a longer intracellular CT domain, which regulates and targets its interaction with macromolecules.^22^ Unlike Cxs, Panx has no sequence homology, but they can form channel proteins in a similar way, and both their NT and CT face the cytoplasm,^2^ and share similar structural features: they both have 4 α-helices TMD, 2 extracellular loops, 1 intracellular loop, 1 intracellular NT segment, and 1 intracellular CT segment^22^ (Fig. 1).

### Panxs formed channels

**Fig. 2:**
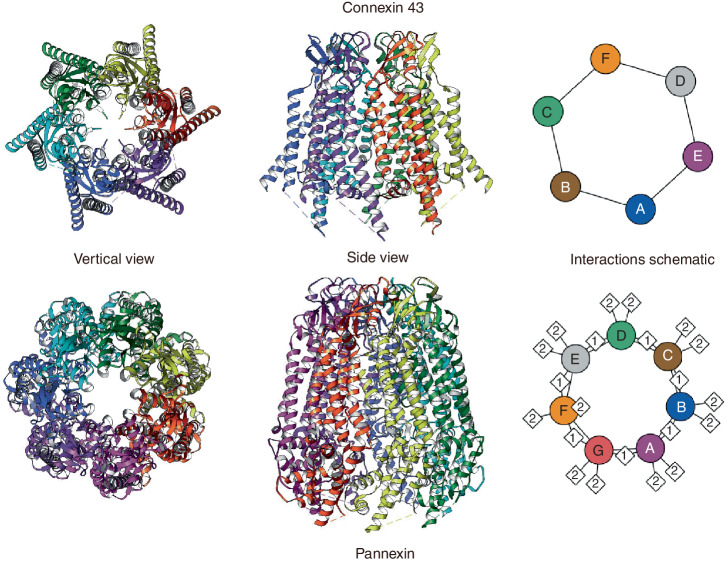
Structure of Panxs and Cxs formed hemichannels. In this pattern diagram, since the respective structures of Panxs and Cxs are similar, we use the structural diagrams of Cx43 and Panx1 as representatives for display

The channels formed by Panxs are primarily HCs. HC mainly exists on the single-layer membrane and is responsible for communicating between the inside and outside of the cell. So far, the jury is still out on whether Panxs can form GJs. Taking Panx1 as an example, there have been many reports that it does not form GJ.^45,46^ Previous studies have generally suggested that Panxs form complexes similar to Cx HCs.^47–49^ But there is also evidence that all three Panxs are able to form GJs between cells.^30,50^ Furthermore, some studies have found that Panxs can form both HCs and GJs in bone, and can even function as unique Ca^2+^ channels in the endoplasmic reticulum (ER).^30,51^ Since the possibility of Panxs forming GJs cannot be completely denied, we will still include discussions related to GJs in subsequent descriptions. GJs facilitate signal transmission and transduction between cells, coordinating cellular responses and regulating physiological functions. GJs can also allow the exchange of ions and small molecules (such as ATP, Ca^2+^, and IP3) between cells and the extracellular matrix or between adjacent cells. GJs are formed by the interaction of two HCs in adjacent cells, with each HC composed of six Cxs on the plasma membrane surface (Fig. 1).^18,52,53^ In contrast to the hexameric structure of Cxs,^39,54–58^ the Panx hemichannel is a heptameric channel^34,36,38^ (Fig. 2). And according to the research conclusions of Bruzzone et al., Panxs may be similar to Cxs and can also form heteromeric channels (Panx3 has not yet been confirmed to participate in the formation of heteromeric channels).^50^

### Basic functions of Panx-channel

Panx channels are involved in the transport of important physiological molecules, such as ATP, intracellular Ca^2+^, glucose, and dye uptake, across membranes.^59–62^ These channels can be activated in various ways. For example, Panx channels can release ATP by interacting with purinergic receptors (P2 receptors), including P2X7 receptors, and can be activated by membrane depolarization and mechanical stretch (Current evidence suggests that Panx1 is not directly activated by membrane stretch, but relies on Piezo1 Channel activation and submembrane increase in Ca^2+^ signal).^11,63^ As a result, Panx channels are associated with a wide range of cellular physiological and pathophysiological functions.^17,64^

Studies demonstrated that Panxs participate in various biological processes, including inflammation,^8,65^ ATP signaling, long-range Ca^2+^ wave propagation,^66^ synaptic plasticity regulation,^67^ vascular homeostasis,^68^ and neurotoxicity.^7^ Additionally, Panxs may contribute to tumor suppression, ischemic cell death, atherosclerosis, apoptosis,^11,69^ human immunodeficiency virus (HIV), and epileptic seizures.^7^ Panxs also play a crucial role in immune function, and cleaving the CT of Panx1 is a means to activate channel opening. The interaction between Panxs and P2X7 receptors stimulates the release of the pro-inflammatory cytokine interleukin-1β through ATP receptor activation, subsequently activating caspase 1.^70^ Panxs can also clear apoptotic cells through ATP and uridine triphosphate (UTP) release.^71^ Furthermore, Panxs can recognize bacterial molecules delivered from endosomes to the cytoplasm and trigger the Toll-like receptor-independent inflammasome.^72^

## Panxs in musculoskeletal system

### Panxs in development of bone

Bone formation involves two highly coordinated processes: endochondral and intramembranous ossification.^73^ Initially, mesenchymal cells derived from the embryonic lineage migrate to the future bone site. Subsequently, these mesenchymal cells differentiate into either chondrocytes, leading to bone formation through endochondral ossification, or osteoblasts, which directly form bone through intramembranous ossification.^74^ The development and maintenance of bone tissue rely on the coordinated actions of osteocytes, osteoblasts, and osteoclasts.

Studies have examined the impact of Panxs on bone density, cortical bone, and diaphyseal structure in Panxs knockout (KO) mice; however, the phenotype of cancellous bone has not been investigated. An in vivo genetic experiment conducted in mice revealed that Panx1 KO had no effect on backbone structure, and intracortical bone resorption did not increase under fatigue load.^75^ Another study demonstrated that Panx3 KO mice exhibited shorter and stronger femoral and humeral diaphyses compared to wild type (WT) mice, with no difference in bone density.^76^ In 2016, the first patient with a homozygous Panx1 variant (c.650 G → A) was reported.^77^ This patient displayed skeletal defects, including kyphoscoliosis, as well as intellectual disability and primary ovarian failure, among other abnormalities. Furthermore, studies have indicated that Panx3 can regulate the proliferation and differentiation of chondrocytes,^61^ osteoblasts,^30^ and osteoprogenitor cells.^78^

#### Panxs in osteocytes

Osteocytes are the most abundant cells in bone tissue and are embedded within the bone matrix.^79^ They play a pivotal role in coordinating the balance between bone formation and bone resorption by integrating mechanical loading and hormonal signals.^80–84^ Fatigue micro-injury, estrogen loss, disuse and other factors can lead to osteocytes apoptosis, and conversely, osteocyte apoptosis can also promote fatigue micro-injury, and osteopenia, etc.^83,85–88^ During this process, apoptotic osteocytes release signals that stimulate the expression of osteoclastic factors in neighboring osteocytes. The release of a large amount of ATP from Panx1 channels during osteocyte apoptosis is a key trigger for this osteocyte-bystander signaling.^75,89^ This signaling relies on the release of apoptosis-dependent signaling factors through Panx1 channels and the activation of P2 receptors.^89^ The presence of Panx1 is crucial in this process, as the loss of Panx1 channels prevents the activation of cortical bone remodeling induced by fatigue. Furthermore, Liu et al. discovered that TGF-β1 increases the expression of Cx43 and Panx1 in osteocytes by activating ERK1/2 and Smad3/4 signaling. This process contributes to the formation of GJs in osteocytes and regulates the intercellular communication of osteocytes.^90^ In addition, Panx1 in osteocytes is also involved in the regulation of muscle mass in mice. Aguilar-Perez et al. found that deletion of Panx1 in bone cells increased muscle mass in young female mice but had deleterious effects on muscle strength in male mice. Researchers propose that the function of Panx1 in osteocytes is dually age- and sex-dependent.^91^

#### Panxs in osteoblasts

Pre-osteogenic cells derived from mesenchymal stem cells have the ability to differentiate into osteoblasts. This differentiation process is regulated by various growth factors, including RUNT-related transcription factor 2 (Runx2), osterix (Osx), osteopontin (OPN), osteocalcin (OCN), and bone morphogenetic protein 2 (BMP2).^92–95^ BMP2 plays a crucial role in inducing the expression of Runx2 and Osx, which are two major transcription factors involved in osteoblastogenesis.^96^ This activation of Runx2 and Osx leads to the expression of downstream osteogenic marker genes, ultimately promoting the terminal differentiation of osteoblasts.^97–99^ The interaction between Runx2 and Smad, a key component of BMP2 signaling, is responsible for the BMP2-induced osteoblastogenesis.^100,101^

Panx3, which is highly expressed in bone and perichondrium/periosteum in the growth plate, including preosteoblasts and osteoblasts,^61^ plays a role in osteoblast differentiation. The expression of Panx3 is promoted by the transcription factor Runx2, but the endogenous expression of Panx3 is not affected in Runx2-Cre transgenic mice.^102–104^ Panx3 expression increases during osteoblastogenesis, and its overexpression can activate the expression of Sp7/Osx and osteocalcin, thereby promoting osteogenic differentiation.^30^ Studies have shown that Panx3 is upregulated during osteoblast differentiation in various cell models, such as C2C12 cells, primary calvarial cells, and MC3T3E1 preosteoblasts.^19,30^ However, the activation of Panx3 alone is not sufficient to initiate osteoblast differentiation, as signals from BMP2 or β-glycerophosphate and ascorbic acid are also required.^19,30^ In addition, both ex vivo and in vitro studies have demonstrated that Panx3 overexpression enhances osteoblastogenesis and bone length, while knockdown of Panx3 inhibits osteoblastogenesis and differentiation.^30,105^ Panx3 has been shown to promote mitogen-activated protein kinase signaling (MAPK) and Wnt/β-catenin pathway activation during osteoblast differentiation. The Wnt/β-catenin signaling pathway can also positively regulate Panx3 expression, suggesting a reciprocal relationship between Panx3 and Wnt signaling.^105^ However, the downstream effects that promote Wnt activation may be inhibited by other factors during osteoblast differentiation.^105^

**Fig. 3:**
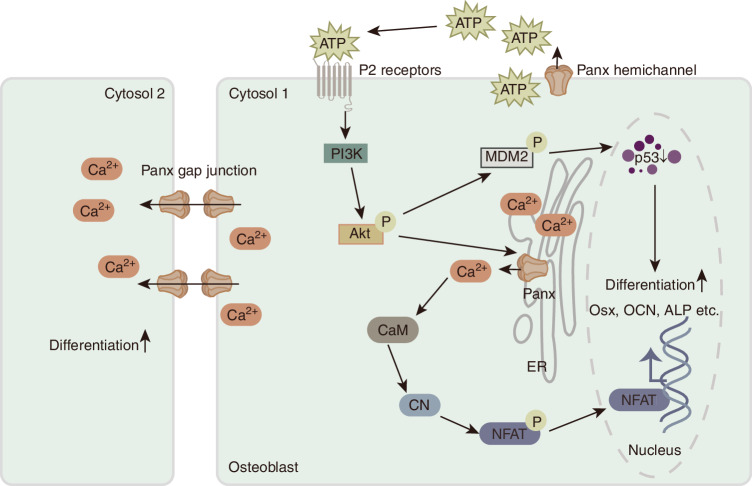
Panx participates in signaling pathways in osteoblast differentiation. Panx3 HCs release intracellular ATP into the extracellular space, where ATP subsequently binds to P2 receptor in an autocrine or paracrine manner. The combination of ATP and P2 receptor activates PI3K/Akt signaling, which in turn activates the Panx3 ER Ca^2+^ channel to release Ca^2+^. Upon Ca^2+^ binding, CaM activates the CN signaling pathway. CN-mediated dephosphorylation activates the NFATc1 in T cells. Activated NFATc1 subsequently translocates into the nucleus and promotes Osx, ALP and OCN. P2 receptor can also activate the Akt/MDM2 pathway through PI3K to promote the degradation of the osteoblast differentiation inhibitor p53. In addition, intracellular Ca^2+^ spreads to neighboring cells through Panx to promote osteoblast differentiation. (HCs hemichannels, ATP adenosine triphosphate, PI3K phosphatidylinositol 3-kinase, ER endoplasmic reticulum, CaM calmodulin, CN phosphatase calcineurin, NFATc1 nuclear factor of activated T cell calcineurin independent 1, ALP alkaline phosphatase, MDM2 mouse double minute 2 homolog, Osx Osterix, OCN osteocalcin, NFAT nuclear factor of activated T cells.)

**Fig. 4:**
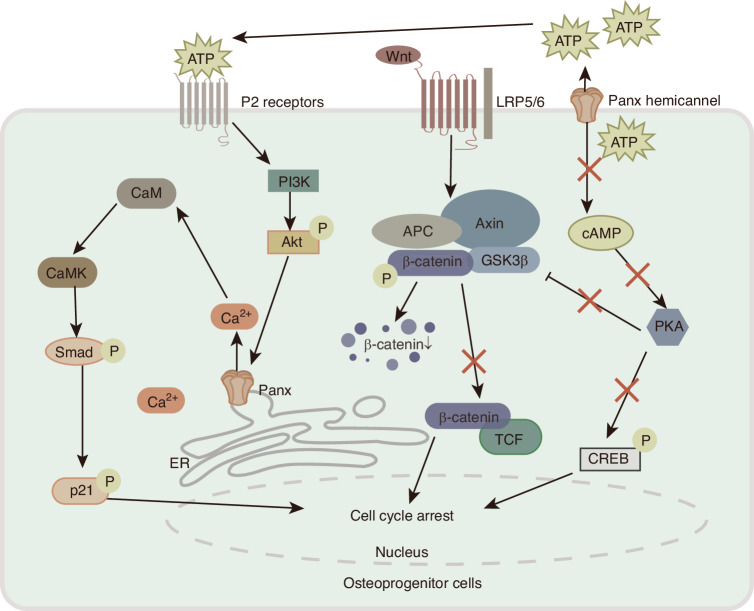
Panx is involved in the signaling pathways of osteoprogenitor cell proliferation and differentiation. Panx HCs release intracellular ATP, which inhibits PKA/CREB signaling and reduces intracellular cAMP content. Inactivation of PKA activates GSK3β, leading to degradation of β-catenin. Wnt/β-catenin signaling is subsequently inhibited and CREB activity is also reduced, thereby inhibiting cell proliferation. In addition, extracellular ATP activates PI3K/Akt signaling, stimulating the opening of Panx3 ER Ca^2+^ channels, thereby activating CaM/CaMK signaling, leading to the activation of Smad and cell cycle inhibitor p21, thereby promoting cell cycle exit. (HCs hemichannels, PKA protein kinase A, cAMP cyclic adenosine monophosphate, CREB cAMP response element binding, GSK3β glycogen synthase kinase 3β, PI3K phosphatidylinositol 3-kinase, ER endoplasmic reticulum, CaM calmodulin, CaMK calmodulin kinase, APC adenomatous polyposis coli, LRP5/6 low-density lipoprotein receptor-related proteins 5 and 6, TCF T cell factor, ATP adenosine triphosphate.)

Panx3 channels not only serve as direct channels between intracellular and extracellular spaces, but they may also act as calcium channels in the ER, with their function relying on the Akt signaling network. ATP can be released into the microenvironment in an autocrine or paracrine manner through Panx3 and bind to purinergic receptors to activate the phosphatidylinositol 3-kinase (PI3K)/Akt signaling pathway. Furthermore, this process can help open Panx3 ER Ca^2+^ channels, resulting in the release of Ca^2+^ from the ER lumen into the cytoplasm. Increased intracellular Ca^2+^ can bind to calmodulin (CaM) and further activate downstream signaling molecules, such as calmodulin kinase II (CaMKII) and the phosphatase calcineurin (CN). When CN is dephosphorylated, it further activates the nuclear factor of activated T cell calcineurin-independent 1 (NFATc1) transcription factor, resulting in NFATc1 nuclear translocation.^30,78,106^ Activated NFATc1 can promote the expression of osteogenic genes and osteogenic markers, such as Osx and alkaline phosphatase (ALP).^30,107–109^ In addition, the overexpression of Panx3 can phosphorylate Akt through the P2 receptor/PI3K pathway, thereby inducing mouse double minute 2 homolog (MDM2) and promoting the degradation of p53 (a negative regulator of osteoblast differentiation)^30,109,110^ (Figs. 3 and 4). These findings suggest that Panxs play critical roles in osteoblast differentiation, although Panx1 and Panx2 have not been extensively studied compared to Panx3.

#### Panxs in osteoprogenitor cells

Like promoting osteoblast differentiation, Panx3 induces osteoprogenitor cells to switch from a proliferation trend to a differentiation trend by utilizing multiple signaling pathways (Fig. 3). Panx3 promotes osteoprogenitor cell cycle arrest at the gap 0/gap 1 (G0/G1) phase by inhibiting corresponding cell cycle molecules such as retinoblastoma protein and cyclin D1.^78^ As previously mentioned, Panx3-mediated ATP release and inhibition of the Wnt/β-catenin pathway can further inhibit cell growth,^51,78^ while Panx3-mediated activation of the Akt pathway can increase Smad1/5 signaling and the level of the cell cycle inhibitor p21.^78^ Interestingly, Ser68 phosphorylation of Panx3 only affects osteoprogenitor cell differentiation but not proliferation, whereas disruption of the putative phosphorylation site Ser303 inhibits both proliferation and differentiation.^111^ Additionally, Panx3 hemichannel inhibits osteoprogenitor cell proliferation by promoting β-catenin degradation through activating glycogen synthase kinase 3-β (GSK3β), and promotes cell cycle exit by increasing the activity of the cell cycle inhibitor p21, thereby facilitating the transition of osteoblasts from proliferation to differentiation.^78,112,113^ These signaling cascades work together to cause osteoprogenitor cells to exit the cell proliferation cycle and differentiate into osteoblasts.^78^

#### Panxs in osteoclasts

Osteoclasts are a resident bone cell type. The function of Panx channels may impact their differentiation (Fig. 4). Ishikawa et al. demonstrated that the expression of osteoclasts and osteoclast differentiation markers decreased in the bones of *Panx3*^*−*^^*/*^^*−*^ mice.^51^ Osteoblasts can regulate the differentiation of bone resorption cells through receptor activator of NF-κB ligand (RANKL) and osteoprotegerin (OPG). Co-culture experiments with osteoblast progenitors and osteoclast progenitors from WT and *Panx3*^*−/−*^ calvaria revealed a decreased level of osteoclast differentiation in the *Panx3*^*−*^^*/*^^*−*^ group compared to the WT group.^51^ This suggests that Panx3-mediated osteoblast differentiation may regulate osteoclast differentiation.^51^ In Panx1 KO mice, the increase in RANKL in the vicinity of apoptotic osteocytes following micro-injury stimulation is attenuated. RANKL is a cytokine required for osteoclast differentiation, indicating that Panx1 may be involved in osteoclast differentiation.^114^ McCutcheon et al. found that both female and male Panx1-deficient mice had significantly reduced cancellous bone in the distal femur and lumbar spine, with higher osteoclast activity observed in female Panx1-deficient mice, while there was no change in males.^115^ Conversely, Panx1-deficient mice exhibited higher osteoclast differentiation and in vitro osteoclast bone resorption activity.^115^ This evidence suggests that alterations in the osteoclast secretome lead to reduced osteoblast function and low bone mass in male Panx1-deficient mice.^115^

### Panxs in cartilage, ligaments and tendons

Currently, Panxs are only known to be expressed in cartilage, ligament, and tendon tissues, however, their function in these cells and how it affects cell function remains poorly understood.

#### Panxs in cartilages

**Fig. 5:**
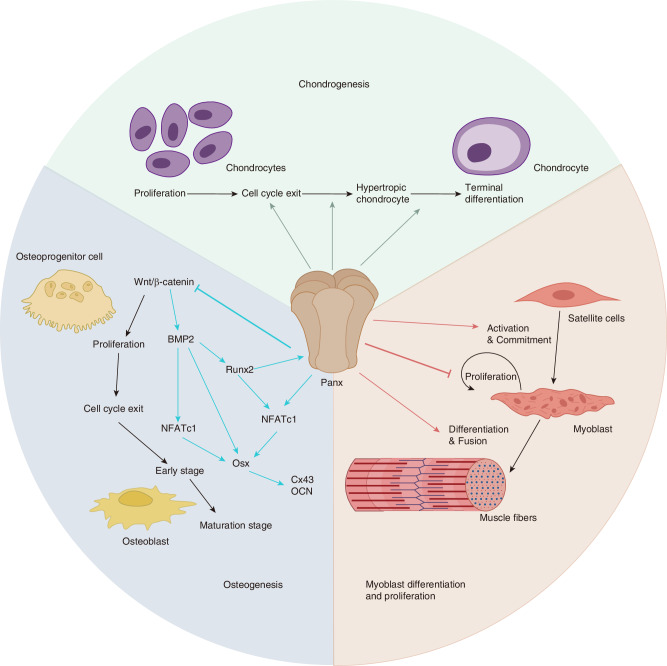
Panxs regulate chondrogenesis, osteogenesis, and myoblast proliferation and differentiation. (Osx Osterix, BMP2 Bone morphogenetic protein 2, NFATc1 Nuclear factor of calcineurin-dependent 1.)

Panxs are involved in regulating the transition between proliferative chondrocytes, prehypertrophic chondrocytes, and terminally differentiated hypertrophic chondrocytes.^19,61^ Transfecting Panx3 promotes chondrogenic differentiation of ATDC5 and N1511 cells, whereas inhibition of endogenous Panx3 impedes differentiation. Furthermore, Panx3 promotes ATP release from the chondrocyte’s intracellular to extracellular space, subsequently inhibiting parathyroid hormone-mediated cell proliferation, intracellular cAMP levels, and phosphorylation of cAMP response element binding (CREB) family transcription factors (Fig. 5). These findings indicate that Panx3 can regulate the transition from proliferation to differentiation of chondrocytes.^61^

**Fig. 6:**
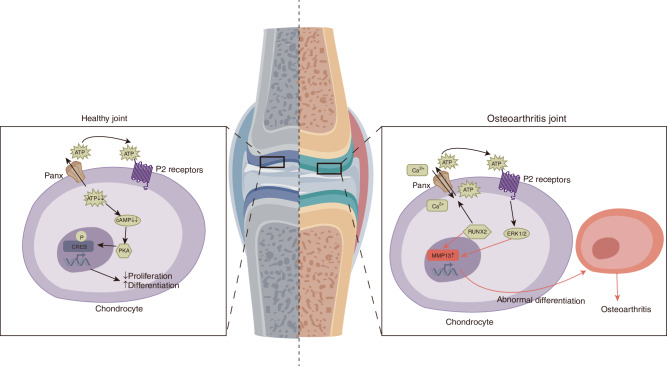
The role of Pannexin channels in healthy and arthritic joints. In healthy cartilage, chondrocytes mediate ATP release through Panx3, and then intracellular ATP reduction activates phosphokinase A and CREB phosphorylation. In arthritic diseases, Panx3 activates P2 receptors through Runx2-mediated ATP release in chondrocytes. Replicate cascades leading to ERK1/2 and MMP13-mediated signaling. Abnormal differentiation of chondrocytes into a hypertrophic chondrocyte phenotype ultimately leads to arthritis. (ALP alkaline phosphatase, cAMP cyclic adenosine monophosphate, CREB cAMP response element binding, MMP13 matrix metalloproteinase 13, ERK1/2 extracellular signal-regulated kinase 1/2, PKA protein kinase A, RUNX2 Runt-related transcription factor 2.)

Notably, Panx3 deficiency in mice disrupts the normal progression of chondrogenesis while not affecting the initiation of hypertrophic chondrocyte differentiation (Fig. 6). This disruption leads to chondrocyte proliferation, prolongation of the prehypertrophic zone, and disorganization of the hypertrophic and terminal chondrocyte layers.^51,116^ However, a study using a chick embryo model yielded inconsistent results: The overexpression of Panx3 did not disrupt chondrocyte arrangement in the avian growth plate, and there were no differences in cartilage histology, chondrocyte proliferation, and hypertrophic markers after a knockdown of Panx3.^117^ Obviously, there are inter-species differences between the avian model of chicken embryo gene knockout or ectopic expression and the mouse gene knockout model. However, in terms of the applicability of the research results to human diseases, the mouse gene knockout model may be more credible.

#### Panxs in ligaments and tendons

In periodontal ligament cells, mechanical strain stimulation causes Panx1 to interact with P2X7 receptors, resulting in the extracellular release of ATP through Panx1 channels.^28^ This interaction between Panx1 and P2X7 receptors may also play a role in the cellular vesicle secretion of interleukin 1β.^28^ However, the detailed molecular mechanism underlying this process remains unclear. Thus far, there have been no reports investigating the role of Panx proteins in ligaments or tendons at the in vivo level. Although in vivo animal studies involving the KO of Panxs have not reported significant abnormalities in these tissues, it is still possible that Panxs may influence the development or function of these tissues.^103^

### Panxs in skeletal muscles

Recently, there has been a focus on studying Panxs in skeletal muscle. However, the potential functions of Panxs in the differentiation and proliferation of skeletal muscle cell have not been thoroughly evaluated yet. So far, only Panx1 and Panx3 in skeletal muscle have been investigated.

In a study conducted by Langlois et al., it was found that Panx1 and Panx3 exhibited differential expression in fetal and adult skeletal muscle tissues. Moreover, they were differentially regulated during the proliferation and differentiation of skeletal muscle myoblast.^25^ Initially, Panx1 levels were observed to be very low in undifferentiated “human primary skeletal muscle cells and myoblasts” (HSMM). However, during the differentiation, the expression of Panx1 increased significantly, becoming the predominant Panx type expressed in differentiated cells. On the other hand, Panx3 showed high expression in adult skeletal muscle but was found to be very low in fetal tissue as well as undifferentiated myoblasts.^25^

It is known that the transformation of pluripotential mesodermal or satellite cells into proliferative myoblasts requires myogenic commitment, which is facilitated by an increase in intracellular free Ca^2+^ concentration ([Ca^2+^]i).^118^ Despite being expressed in small amounts in non-differentiated myoblasts, Panx1 plays a crucial role in myogenic commitment.^119^ Panx1 is predominantly localized in the T-tubules of fully differentiated myofibers, where it forms Panx1 HCs that release ATP into the extracellular medium.^24,120^ The released ATP then activates P2 receptors, leading to an elevation in [Ca^2+^]i and subsequent enhancement of muscle contraction.^24^ This response is absent in muscles of *Panx1*^*−/−*^ mice and can be blocked by Panx1 channel inhibitors.^24^ During skeletal muscle contraction, serine and threonine protein kinases, such as CaMKII, PKA, and PKC, are activated,^121^ resulting in the phosphorylation of serine and threonine residues in the CT domain of Panx1. Additionally, repeated electrical stimulation of muscle fibers promotes the phosphorylation of Panx1.^24^ Consequently, electrical stimulation of skeletal muscle myotubes can induce the opening of Panx1 channels, ATP release, and activation of plasma membrane P2X (ionotropic) and P2Y (metabotropic) receptors, thereby modulating both Ca^2+^ homeostasis and muscle physiology.^26^ These findings collectively suggest that Panx1 is involved in muscle plasticity and influences muscle strength. Mechanistically, Suarez-Berumen et al. demonstrated that Panx1 activates lipid-based signaling pathways, coordinating myoblast activities necessary for skeletal muscle regeneration.^122^ They observed that Panx1 activation of P2 receptors mediates lipid signaling cascades in myoblasts, supporting myoblast migration and fusion. Furthermore, Panx1 regulates the interaction between myoblasts and the extracellular matrix by inducing ADAMTS proteins, facilitating extracellular matrix remodeling.^122^ However, the specific role of Panx1 phosphorylation in muscle contraction and potentiation remains unclear, and the protein kinase responsible for mediating this effect has yet to be identified.

Recently, a unique sex-dependent function of Panx1 has been discovered in skeletal muscle.^123^ In global Panx1 KO male mice, muscle fiber size and strength, as well as the number of satellite cells (SCs), are reduced. Additionally, early SC differentiation and myoblast fusion are also altered in these male mice. However, no such effects were observed in Panx1 KO female mice. Interestingly, although Panx1 KO mice show an increased number of regenerated fibers after acute injury, these newly formed fibers are smaller in male mice.^123^ These findings indicate that Panx1 plays a crucial role in regulating muscle development, regeneration, and the number of satellite cells in mice, with notable sex differences.

The pathways regulating Panx3 expression during myogenesis remain unclear. However, the activation of the Toll-like receptor 4 (TLR4)/nuclear factor-κB (NF-κB) pathway in L6 myotubes can significantly increase Panx3 expression.^33^ Moreover, when ectopically expressed in HSMM, Panx3 inhibits cell proliferation.^25^ Additionally, Panx3 overexpression promotes the differentiation and fusion of HSMM, as evidenced by an increased percentage of myosin heavy chain-positive HSMM and multinucleated cells.^25^ Notably, undifferentiated HSMM express a high level of an approximately 70 kD immunoreactive species of Panx3, which is dramatically downregulated during differentiation. Knockdown of this species significantly reduces HSMM proliferation.^25^ These findings suggest that Panx3 species may play a crucial role in maintaining the differentiated and nonproliferative state of skeletal muscle. However, it is important to exercise caution when interpreting these results due to the uncertain specific mechanisms mentioned earlier.

### Panxs in musculoskeletal system disease

#### Panxs in osteoarthritis

Osteoarthritis (OA) is a prevalent degenerative joint disease characterized by a combination of inflammatory and metabolic factors.^124^ Prominent manifestations of OA include progressive pain, joint swelling, and limited mobility.^125^ It affects the entire joint, leading to pathological changes in bones and soft tissues such as the synovium, meniscus, and ligaments. However, cartilage loss and the inability to repair damaged cartilage remain significant pathological features of OA.^126^

Panxs are associated with OA and have distinct molecular mechanisms and roles in the pathobiology of primary and secondary OA development.^127^ (Fig. 6) In a study of surgically induced OA in rats, it was found that Panx3 mRNA was significantly increased in osteoarthritic cartilage compared with controls.^127^ The expressions of matrix metalloproteinase 13 (MMP13) and Panx3 were upregulated in the cartilage degeneration area caused by surgery for medial meniscal instability in WT mice, but were not observed in the cartilage of sham-operated controls.^128^ Additionally, Panx3 expression is upregulated in human weight-bearing osteoarthritic cartilage compared with non-weight-bearing controls.^129^

Compared with controls, chondrocyte-specific Panx3 KO mice had milder OA symptoms, showing mostly normal joints, and reduced proteoglycan loss and cartilage degeneration.^76,129^ Interestingly, another study showed that Panx3 deletion had an opposite, more deleterious effect on primary OA: aged (18–24 months old) Panx3 KO mice exhibited full-thickness articular cartilage erosion, increased osteophyte size, and low-grade synovitis in their knees, whereas WT knees exhibited minimal cartilage damage.^130^ Therefore, it can be explained that Panx3 deficiency accelerates the progression of OA during aging but has a chondroprotective effect on post-operative OA in young mice.^129,130^

In cell and animal models of temporomandibular joint osteoarthritis (TMJOA), the expression of Panx3, P2X7R, and cartilage matrix degradation-related enzymes increased, and inflammation-related pathways were activated, leading to the release of ATP from intracellular to extracellular compartments.^131^ However, in TMJOA rats, the deletion of Panx3 reduced condylar cartilage injury and hindered the increase of P2X7R, cartilage matrix degradation-related enzymes, and NLRP3 in the condylar cartilage tissue. On the other hand, overexpression of Panx3 enhanced these responses, which could be reversed by silencing Panx3. Additionally, the regulation of Panx3 overexpression was reversed by a P2X7R antagonist. Therefore, it can be inferred that Panx3 may activate P2X7R by releasing ATP and contribute to inflammation and cartilage matrix degradation in TMJOA.^131^

#### Panxs in intervertebral disc degeneration

Intervertebral disc (IVD) degeneration is a common spinal disease and a frequent cause of low back pain, with its prevalence increasing with age. The degeneration of IVD involves progressive structural changes in the disc, along with significant alterations in metabolic homeostasis.^132^

Similar to its role in OA,^129,130^ Panx3 exhibits distinct functions in age-related primary IVD degeneration and injury-induced secondary IVD degeneration. In normal aging mice, there were no significant differences in histopathological scores, chondrocyte hypertrophy, and extracellular matrix between WT and Panx3 KO lumbar IVDs, indicating that Panx3 deletion did not affect primary IVD degeneration. However, in a model of injury-induced IVD degeneration, the detrimental effects of Panx3 on IVD were evident in Panx3 KO mice, as evidenced by increased structural integrity of the annulus fibrosus (AF), reduced mast cells, and increased average AF lamellar thickness.^133^ Importantly, it was observed that in Panx3 KO mice, the uninjured IVD adjacent to the acupuncture site exhibited accelerated degeneration of the nucleus pulposus, while the adjacent IVD in WT mice remained completely healthy.^133^ This suggests that mechanosensitive Panx3 channels may participate in IVD homeostasis mechanisms, regulating the altered biomechanics of adjacent healthy joints.

#### Panxs in Duchennes muscular dystrophy

Duchennes muscular dystrophy (DMD) is a severe and common muscle disease characterized by X-linked mutations in the dystrophin gene. This genetic mutation leads to the loss of dystrophin, making muscle fibers more susceptible to mechanical damage and impairing satellite cell activation and muscle fiber regeneration. Patients with DMD experience progressive muscle atrophy, adipocyte infiltration, and ultimately suffer from paralysis and death.^134,135^

Panx1, a protein, has been found to play a role in excitation-transcription coupling in skeletal muscle as part of a multi-protein complex that includes dihydropyridine receptors, P2Y2 receptors, and caveolin-3.^136^ Interestingly, this complex also interacts with dystrophin.^136^ Mdx mice, which carry mutations in the dystrophin gene, are commonly used as animal models for DMD.^134^ In mdx mice, Panx1 expression levels are higher in myofibers compared to control mice,^137^ and there is an increased release of ATP through Panx1 channels.^138^ While exogenous ATP has anti-apoptotic effects on normal skeletal muscle fibers, it activates pro-apoptotic pathways in myofibers from mdx mice.^138^

A study using mouse models of mild and severe DMD (dystrophin-deficient and dystrophin/dystrophin double KO, respectively) found significantly reduced levels of Panx3 in the dystrophic muscles of these mice, suggesting dysregulation of Panx3 expression in DMD.^139^ Based on these findings, targeting Panx1 channel activity to reduce ATP release may hold potential for benefiting DMD patients, although further research is needed to confirm this. Additionally, more investigation is required to understand the levels and potential dysfunction of Panx3 in mdx fibers.

## Perspective of Panxs in musculoskeletal system

### Association with Cx43 in musculoskeletal system

Cx43 is the most highly expressed Cx isoform in the musculoskeletal system and is considered one of the most important Cxs. It is found in various bone cells, synovial tissue, cartilage, and other tissues, and plays a pivotal role in the musculoskeletal system^140^ (Table 1). Panx proteins are also highly expressed during musculoskeletal system development and are considered major GJ proteins.^141^ Interestingly, Panx channels can be blocked by several Cx hemichannel and channel inhibitors, such as carbenoxolone. This suggests that Panx channels may share a common gating mechanism and similar physiological functions with Cxs.^141^

**Table 1:** Panxs and Cxs in musculoskeletal system

Cx43			Panx3			Panx1		
Cell type	Process	Ref.	Cell type	Process	Ref.	Cell type	Process	Ref.
Osteoblast	Differentiation (early stage)	^187–191^	Osteoblast	Differentiation	^192^	Osteoclast	Differentiation	^114^
	Cell survival	^193–196^		Mineralization	^30^		Bone resorption activity	^115^
	Mineralization	^197–200^		ER Ca^2+^ releasing	^30,78,106^	Periodontal ligament cell	Mechano-sensation	^103^
Osteoclast	Differentiation, preosteoclast fusion, survival	^201–204^	Osteoprogenitor cell	Differentiation	^78,112,113^	Myoblast	Differentiation	^25^
	Osteoclastic bone resorption	^202,205^	Osteoclast	Differentiation	^51^		Migration and fusion	^122^
Osteocyte	Mechano-sensation	^206–210^	Chondrocyte	Differentiation	^19,61^	Fully differentiated myofiber	T-tubule releasing ATP	^24^
Myoblast	Transducing biophysical signals	^140^	Myoblast	Differentiation	^25^			
	Cell activities and lifespan	^191,205,211–213^						
Synovial macrophage-like type A cells	Inflammation	^214–217^						
Chondrocyte	Mechanotransduction	^140^						
	Cell-to-cell communication	^140^						
	Proliferation and biosynthesis	^218^						

There is evidence that Cx43-KO and Panx3-KO mice develop bone abnormalities. In fact, Cx43-KO mice were found to have abnormal bone development during the embryonic period, and these mice died after birth.^142^ Roberto Civitelli’s group further found that lack of Cx43 resulted in delayed endosteal and endochondral ossification. Specifically in the skull, osteoblast abnormalities and known defects in neural crest cell migration combine to cause craniofacial defects and patent foramen.^143^ In contrast, Panx3-KO mice have no other obvious abnormalities except for shortened long bone length.^76^ Compared with Cx43-KO mice, Panx3-KO mice showed obvious bone abnormalities during the neonatal period, and Cx43 expression was reduced in the limbs and skull; however, Panx3 expression was normal in Cx43-KO mice. Notably, the body size of Panx3- and Cx43-double KO mice was similar to that of Panx3-KO mice.^116^ These results suggest that the effect of Panx3 may take precedence over the effect of Cx43. In fact, Panx3 can serve as an upstream regulator of Cx43. It can regulate the expression of Cx43 through the Wnt/β-catenin signaling pathway and the Osx pathway.^144^ During the osteoblast proliferation stage, Panx3 promotes β-catenin degradation by activating osteocyte GSK3β, thereby inhibiting the Wnt/β-catenin signaling pathway and cell proliferation.^78^ As immature osteoblasts differentiate into mature osteoblasts, the expression of Panx3 gradually decreases.^105^ This leads to the synthesis of β-catenin mRNA during osteoblast development, resulting in the recovery of Wnt/β-catenin signaling and increased Cx43 expression.^51,145^ Additionally, Panx3 can upregulate intracellular Ca^2+^ levels to induce the expression of Osx, thereby activating the CaM/NFAT pathway and increasing Cx43 expression.^30,51,146^ Functional similarities and differences in the musculoskeletal system between Panx3 and Cx43 have also been recognized. Panxs function through ATP HCs, ER Ca^2+^ channels, and GJs to transfer intracellular Ca^2+^ to neighboring cells and the extracellular environment. In contrast, Cx43 channels currently have no evidence of functioning as Ca^2+^ channels in the ER and have only been found to have hemichannel and GJ activities on the cell membrane,^51^ which is a major functional difference between the two. Previous studies have shown that Panx3, but not Cx43, localizes to the ER and functions as an ER Ca^2+^ channel.^51,147^ The specific mechanism underlying this functional difference may originate from the structural differences between Panx3 and Cx43, although it is not yet clear.

In this article, we have discussed the presence of Panx in the musculoskeletal system and its physiological functions. We have also explored its relevance in the development of musculoskeletal diseases such as OA and IVD degeneration. The major cell types found in joints, including osteoblasts, osteoclasts, osteocytes, and chondrocytes, express one or more isoforms of Panx. In joint diseases, the expression levels of Cx43, another protein involved in GJs, increase in bone, cartilage, and synovial tissues during disease and inflammatory episodes.^147–150^ Most studies have indicated that inhibiting the reduction of Cx43 in joint tissues could be beneficial in preventing the occurrence and progression of joint diseases, considering the inflammatory component of their pathology and the role of matrix metalloproteinases (MMPs).^151,152^ On the other hand, Panx1 is involved in the pathological response of cartilage stiffness and mediates joint pain.^147,153,154^ Panx3 is implicated in cartilage damage in both mouse and human OA^155^ and promotes hypertrophic chondrocyte differentiation.^50,61^ Since chondrocytes in OA patients exhibit a hypertrophic-like phenotype, and pain is a major factor leading to disability in joint diseases,^156^ it is an attractive target to consider combining Cx43 and Panxs-targeted drugs when developing treatments for joint diseases, aiming to slow disease progression and reduce pain.^157^ Moreover, the Panx1/P2X7 receptor complex function and ATP release act as a “find me” signal, necessary for macrophage recruitment,^158^ osteocyte apoptosis, and enhanced bone resorption.^65,159^ This suggests that Panxs could be potential preventive and therapeutic targets for bone lesions such as osteoporosis.^160^ However, to further explore these ideas, we need a better understanding of the similarities and differences in the roles of Panx and Cx GJs in joint tissue and how their interactions impact joint disease.

However, it should be noted that Cx is almost not expressed in mature skeletal muscle cells. The muscle fibers of most innervated skeletal muscles do not contain GJs.^5^ Likewise, Cx HCs are absent in innervated skeletal muscles of adult rodents.^27^ However, the lack of Cx expression in skeletal muscle is not a universal feature of all vertebrates.^161^ In contrast, normal adult muscle expresses Panx1 but not Panx2 or Panx3.^5,24,120^ Panx1 forms HC and is localized in the transverse T-tubule, next to the dihydropyridine receptor,^5^ and participates in skeletal muscle contraction response and glucose uptake.^24^

### Similar treatments on Panxs and Cxs

Treatments targeting Panxs and Cxs exhibit similarities and can even utilize the same drugs (Table 2). Currently, there are five complexes that can be targeted for treatment: Panxs or Cxs GJs and plasma membrane HCs, as well as mitochondrial HCs.^162^ These treatment options include drugs specifically designed for Panxs and Cxs, as well as repurposing of existing drugs. For instance, Carbenoxolone, an anti-gastric ulcer medication, was discovered to block Cx43 channels as early as 1986.^163^ Bruzzone et al. demonstrated its inhibitory effect on Panx1 channels, and it has since been widely used in Panx1 research.^163–165^ The inhibition of Panx1 channels by carbenoxolone exhibits a concentration-dependent response.^62,164^ Recent cryo-EM analysis of Panx1 channels revealed that carbenoxolone triggers allosteric inhibition by clustering in the groove between the extracellular loop 1 and extracellular loop 2 domains of Panx1, thereby stabilizing its closed conformation.^36,166^ Despite its defective selectivity, studies have shown that carbenoxolone-induced inhibition of Panx1 channels attenuates cancer metastasis in mice,^167^ reduces platelet aggregation,^165^ and inhibits the activation of the NLRP3 inflammasome.^168^ Carbenoxolone has also demonstrated protective effects against various types of ischemic injury, such as acute renal ischemia/reperfusion injury,^169^ pulmonary ischemia/reperfusion injury,^170^ and stroke.^171^ Probenecid, a drug commonly used for gout treatment, has been found to be effective in targeting the channels formed by Panxs and Cxs. The mechanism of action of probenecid is similar to that of carbenoxolone, but unlike carbenoxolone, probenecid interacts with the first extracellular loop of the protein and specifically inhibits Panx1 channels at high concentrations.^65^ Tenofovir, an antiviral drug primarily used for treating viral hepatitis, has also been discovered to inhibit Panx1-mediated ATP release.^172^

**Table 2:** Existing Panxs-modulating drugs and drugs targeting Cx43

Target	Drug	Cell/Tissue	References
Cx43-GJ	Meclofenamate	Carcinoma-astrocytes	^219^
Cx43-GJ	Cx43 oligonucleotide	Corneal epithelial cells	^220^
Cx43-GJ	Rotigaptide	Myocardium	^221^
Cx43-GJ	α-Connexin carboxyl terminal peptide	Skin, myocardium	^222,223^
Cx43-GJ	Flufenamic acid	N2A cells	^179^
Cx43-HC	Tonabersat	Human cerebral microvascular endothelial cells	^224^
Cx43-HC	Peptide5	Spinal cord neurons	^225^
Cx43-HC	Gap19	Astrocytes	^226^
Panx1	Probenecid	Xenopus laevis oocytes	^59,227^
Panx1	Tenofovir	Skin	^172^
Panx1 channel	Brilliant Blue FCF	Xenopus laevis oocytes	^228^
Panx1 channel	18α-, 18β-glycyrrhetinic acid	Xenopus laevis oocytes	^164,229^
Panx1 channel	Arachidonic acid	Xenopus laevis oocytes	^230^
Panx1 channel	Palmitic acid	Human and rat liver cell lines	^230,231^
Panx1 channel	^10^Panx1	e.g. Phagocytes, macrophages	^11,232^
Panx1-channel	Carbenoxolone	HEK293 cells	^8,62,179^

So far, only part of the mechanism of action of these inhibitors has been revealed. It is believed that glycyrrhetinic acid (GA) may directly interact with channels when inserted into the cell membrane, thereby binding to channels and causing conformational changes.^173,174^ In addition, changes in the phosphorylation status of channel subunits or reduced expression of subunits are also potential mechanisms for the effects of GA.^175,176^ Carbenoxolone is a more water-soluble derivative of GA,^177^ and its inhibitory mechanism on Panx1 channels has been initially revealed: Carbenoxolone may act on W74 in the first extracellular loop. And when this site is mutated to a nonaromatic residue, Carbenoxolone reverses its inhibitory effect and enhances the voltage-gated channel activity of Panx1.^166^ Similarly, Probencid is also thought to bind to the first extracellular loop of Panx1, further prompting that Panx1 may undergo conformational changes: this includes the bending of the N-terminal region toward the cytoplasm and the changed tilt angle between each subunit and the membrane plane.^178^ In addition, there are many reagents that inhibit the channels formed by Panxs and Cxs, but more inhibitory mechanisms have yet to be explored.^179,180^

In various diseases, targeting Cxs has proven to be an effective therapeutic approach by either permanently or temporarily closing the channels. This strategy holds great potential for systematic research and targeted therapy of Panxs in the future. Peptides that specifically target Cxs can also be utilized for intervention (Table 2). For instance, the peptide α-connexin carboxyl terminus (ACT1), which is used clinically, can localize Cx43 at the GJ site on the cell membrane boundary of breast cancer cells. This localization enhances the functional activity of GJs, leading to impaired proliferation and survival of breast cancer cells.^181^ ^10^Panx1 is a 10-amino acid peptide derived from the extracellular link of the Panx1 protein that selectively inhibits Panx1 without affecting ATP-induced currents.^8^ This peptide is made by simulating the sequence (WRQAAFVDSY) in the second extracellular loop region of Panx1, and may exert an inhibitory effect on Panx1-channel through the side chains of Gln3 and Asp8.^182^ Furthermore, antisense oligonucleotides have emerged as a promising therapeutic modality for targeting channel proteins. Clinical application of Cx43 antisense oligodeoxynucleotides has been successful in treating severe ocular surface burns.^183^ Considering these findings, Panxs-targeted treatment strategies can draw valuable insights from the ideas and approaches employed in Cxs research.

### Panx2, which has yet to make its mark

Additionally, Panx2 is an important member of the Panx family and holds significant potential. While Panx1 and Panx3 have been confirmed to play roles in the musculoskeletal system, Panx2 has not yet been implicated in any cellular activities or pathogenesis. Panx2 is primarily localized in the membrane-binding region within the cytoplasm, whereas other Panxs are predominantly found in the plasma membrane.^184^ Intriguingly, a recent study identified Panx2 as a novel mammalian cell mitochondria-associated membrane protein, suggesting its potential localization at the ER-mitochondria contact site.^185^ This implies that Panx2 may serve as a novel contact protein, connecting the ER and mitochondria. Given the involvement of the ER-mitochondria tethering complex in the regulation of calcium or lipid homeostasis, cell survival, apoptosis, and its importance in degenerative diseases,^186^ Panx2 is likely to exert significant effects in the musculoskeletal system.

## Conclusion

Panxs play a crucial role in various cellular activities within the musculoskeletal system, serving as components of cellular GJ or HCs. However, the study of Panxs is still limited and lacks in-depth exploration. Cxs, which share similarities with Panxs, particularly in certain functions, can provide valuable insights into the intercellular signaling dynamics of Panxs. By combining the knowledge of both Panxs and Cxs, new therapeutic targets can be discovered, and novel treatment strategies can be developed. Manipulating the expression or activity of Panxs and Cxs could optimize the musculoskeletal system and enhance the efficacy of current therapeutic agents. With the aging population and the increasing prevalence of muscle and joint diseases, there is a growing socioeconomic burden and a pressing need for innovative concepts and treatments. We firmly believe that Panxs hold great promise as targeting molecules and warrant further attention and research.
